# Mobulid rays feed on euphausiids in the Bohol Sea

**DOI:** 10.1098/rsos.161060

**Published:** 2017-05-24

**Authors:** Christoph A. Rohner, Katherine B. Burgess, Joshua M. Rambahiniarison, Joshua D. Stewart, Alessandro Ponzo, Anthony J. Richardson

**Affiliations:** 1 Marine Megafauna Foundation, Praia do Tofo, Inhambane, Mozambique; 2 Shark and Ray Research Group, School of Biomedical Sciences, The University of Queensland, St Lucia, Queensland 4072, Australia; 3 Large Marine Vertebrates Research Institute Philippines, Cagulada Compound, Tejero, Jagna, 6308 Bohol, Philippines; 4 Scripps Institution of Oceanography, University of California, San Diego, 9500 Gilman Drive., La Jolla, CA 92093, USA; 5 The Manta Trust, Catemwood House, Corscombe, Dorchester, Dorset DT2 0NT, UK; 6 CSIRO Oceans and Atmosphere, EcoScience Precinct, Brisbane, Queensland 4102, Australia; 7 Centre for Applications in Natural Resource Mathematics, School of Mathematics and Physics, University of Queensland, Brisbane, Queensland, Australia

**Keywords:** feeding ecology, tropical zooplankton, elasmobranchs, Mobulidae, mesopelagic prey, krill

## Abstract

Mobulid rays have a conservative life history and are caught in direct fisheries and as by-catch. Their subsequent vulnerability to overexploitation has recently been recognized, but fisheries management can be ineffective if it ignores habitat and prey preferences and other trophic interactions of the target species. Here, we assessed the feeding ecology of four mobulids (*Manta birostris*, *Mobula tarapacana*, *M. japanica*, *M. thurstoni*) in the Bohol Sea, Philippines, using stomach contents analysis of fisheries specimens landed between November and May in 2013–2015. We show that the mobulids feed heavily on euphausiid krill while they are in the area for approximately six months of the year. We found almost no trophic separation among the mobulid species, with *Euphausia diomedeae* as the major prey item for all species, recorded in 81 of 89 total stomachs (91%). *Mobula japanica* and *M. thurstoni* almost exclusively had this krill in their stomach, while *M. tarapacana* had a squid and fish, and *Ma. birostris* had myctophid fishes and copepods in their stomachs in addition to *E. diomedeae*. This krill was larger than prey for other planktivorous elasmobranchs elsewhere and contributed a mean of 61 364 kcal per stomach (±105 032 kcal s.e., range = 0–631 167 kcal). Our results show that vertically migrating mesopelagic species can be an important food resource for large filter feeders living in tropical seas with oligotrophic surface waters. Given the conservative life history of mobulid rays, the identification of common foraging grounds that overlap with fishing activity could be used to inform future fishing effort.

## 1. Introduction

Mobulid rays are large pelagic planktivorous elasmobranchs comprising 11 species with a global distribution in tropical to warm-temperate waters [1]. Mobulids have a conservative life-history strategy and are caught in direct and indirect fisheries [1–4], making them vulnerable to overexploitation [5,6]. *Mobula japanica* and *M. thurstoni* are listed as ‘Near threatened' on the IUCN Red List, *M. tarapacana* as ‘Vulnerable' and all three species have an unknown population trend, while *Manta birostris* is ‘Vulnerable' with a decreasing trend [1,7–10]. The susceptibility of mobulids to fisheries-induced population declines and the role of international trade in driving targeted mobulid fisheries [7] led to the addition of all mobulid species to CITES Appendix II and CMS Appendices I & II between 2013 and 2016. However, basic biological knowledge is lacking for most mobulid species, making it difficult to assess and manage mobulid fisheries. Trophic knowledge can inform ecosystem-based fishery management [11] and can help reduce by-catch [2] through the identification of critical foraging areas, where the occurrence of target species and fishing activity overlap. For example, if a fishery targets multiple species feeding in the same area, catch rates for the overall assemblage can stay high due to abundant feeding opportunities, but changes in abundance of individual species might vary [12]. The dynamics of multi-species fisheries, therefore, present a major problem for setting any total allowable catches of a particular species, and can hinder overall management across the range of species that are targeted, as it is difficult to predict catch composition [13].

There are few studies reporting high-resolution stomach contents dietary data for mobulids, as is the case for all large planktivorous elasmobranchs. The diet of *M. japanica* and *M. thurstoni* in the Gulf of California, Mexico, has been described in detail, with both species feeding largely on the euphausiid *Nyctiphanes simplex* [4,9]. Many rays from the Gulf of California had empty stomachs, including four of five *M. tarapacana*, with the one full stomach containing fish remains. The museum specimen of *Manta alfredi* caught in 1935 in Australia had mostly large copepods in its stomach [10].

**Figure 1.**
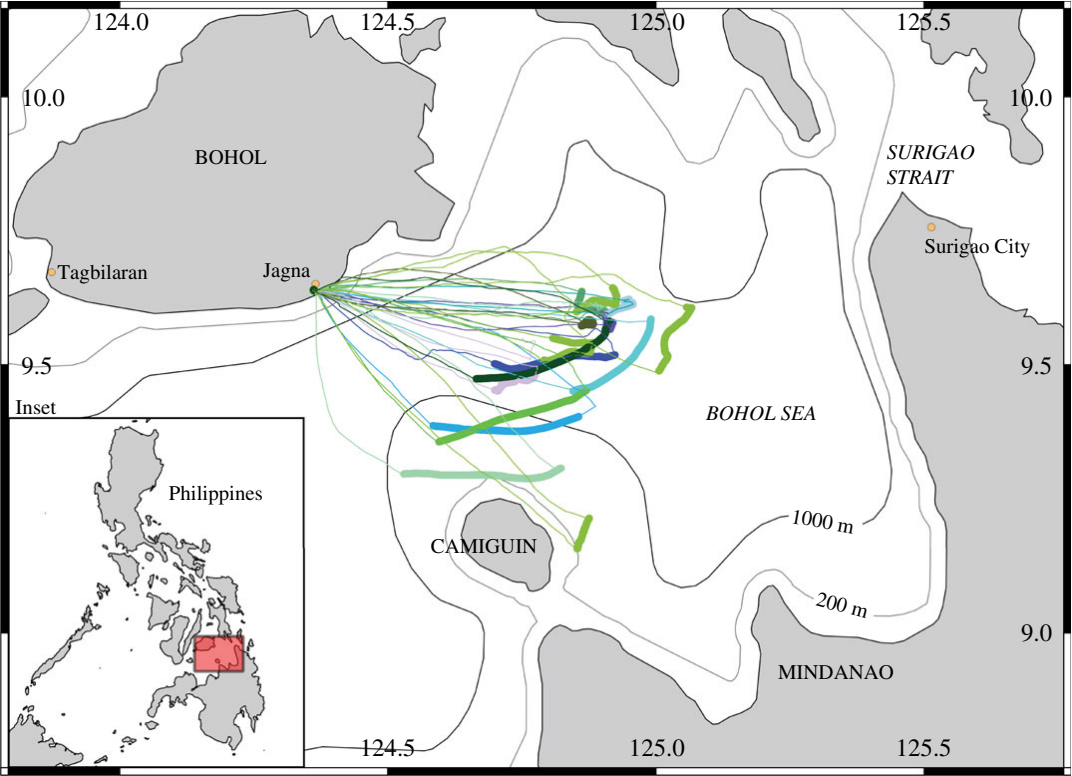
Fishing locations in the Bohol Sea. Study area with 200 m and 1000 m isobaths, and the inset showing the Philippines with our area of interest highlighted. GPS tracks of fishing boats are also included, with thin lines representing the tracks in transit and thick lines showing the trajectories of drifting nets to illustrate the exact fishing locations in the Bohol Sea. Different colours represent different boats.

Recent dietary studies of large planktivores have used non-lethal methods to infer diet because it is unethical to catch these threatened species and access to dead specimens has been rare. For example, biochemical methods require a small tissue sample, collected from live, free-swimming animals and biochemical profiles can then be compared with that of suspected prey [14–17]. Biochemical techniques usually only provide low-resolution, indirect information on an average integrated dietary signal over time and what trophic levels animals feed at, and it is difficult or impossible to conclude which prey species are eaten. Another non-lethal technique for characterizing diet in large planktivores involves the collection of plankton alongside feeding animals. Like stomach contents analysis this can give a short-term view of diet, but it is still considered an indirect technique as it is impossible to determine whether all or only some of the prey items are ingested [18,19]. For a direct, short-term, assessment of diet, access to dead specimens is required, which can be challenging for rare or internationally protected species, such as mobulids. A targeted mobulid fishery operates out of Jagna, Bohol, in the Philippines from November to May (figure 1); [20,21], giving us the opportunity to study the short-term feeding ecology of landed species directly.

The Bohol Sea is a deep, topographically isolated basin connected to the Pacific Ocean by the shallow Surigao Strait (58 m). The ‘Bohol Jet' surface current in the north of the Bohol Sea is the extension of water flowing in at high speeds through the Surigao Strait [22] and is the main oceanographic feature influencing the mobulid fishing ground. The fast inflow of Pacific waters through the Surigao Strait induces entrainment of deep, cold, nutrient-rich waters to the upper layer [22,23]. The intensification of the Bohol Jet during the northeast monsoon (November–April), combined with inflow from the Agusan river, results in high primary productivity and a concurrent plankton bloom [22,23], which coincides with the mobula fishing season. The Bohol Sea also hosts a variety of other large planktivores, including the whale shark *Rhincodon typus* [24] and the blue whale *Balaenoptera musculus* [25].

All mobulids are filter feeders that probably target zooplankton and small fishes, but the prey types and sizes could vary among species due to differences in their anatomy and ecology. Mobulid species found in the Bohol Sea range in size from a 1 m wide *M. thurstoni* to *Ma. birostris* that grows to 7 m wide [1]. Some of the larger species dive deep and spend more time in the mesopelagic zone than smaller species, potentially accessing different prey sources [26–28]. There are also slight variations in gill raker structure and the mechanisms involved with capturing prey among mobulids [29], further suggesting that the different species could have differing diets. Dietary differences among co-occurring mobulid species are thus likely and could be driven by different sizes, dive behaviours and feeding structures.

Here, we examined the short-term feeding ecology of *Manta birostris*, *Mobula tarapacana*, *M. japanica* and *M. thurstoni* over 3 years in the Bohol Sea, Philippines, using stomach contents analyses. We identified prey items with microscopy, calculated a size spectrum for main prey type, estimated prey caloric content, and investigated trophic separation in the short-term diet among the four co-occurring mobulid species. Our results show that vertically migrating mesopelagic zooplankton species can be an important food resource for large filter feeders living in tropical seas with oligotrophic surface waters.

## 2. Material and methods

All stomach samples were collected from a mobulid landing site in Jagna on the island of Bohol in the Philippines (figure 1), where fishers have long targeted mobulids as their main catch [20]. In 2015, seven boats stationed at Jagna regularly fished for mobulids in the Bohol Sea between the landing site and Camiguin Island. Some boats carried a handheld GPS unit (747 A+ GPS Trip Recorder) to track where they fished. We mapped those GPS tracks of boats that made catches used in this study and defined soak time (i.e. when the net was drifting in the water usually for approximately 7 h) as locations with a speed < 5 km h^−1^ (figure 1). Fishing boats left Jagna in the afternoon and returned to the beach the next morning to sell their catch. Mobulids were caught using drifting gill nets that are approximately 1000–2000 m long, 30 m high at between approximately 10 and 40 m depth and have a mesh size of approximately 50–80 cm. *Mobula* spp. were killed and stored whole onboard, while *Ma. birostris* were too large and thus usually dragged to shore alive and still entangled in the net. Rays were processed on the beach at the landing site.

The majority of samples (*n* = 64, 72% of total samples) were collected from February to April 2015, when we removed whole stomachs from the end of the oesophagus to past the pyloric stomach and weighed them prior to removing the contents (table 1). We dissected the stomachs and removed and weighed the contents prior to taking subsamples. Stomach contents appeared homogeneous and we took a subsample of approximately 2.5 ml (equivalent to 2 g) in a 10 ml tube and stored it in 5% formalin. Before discarding the rest of the stomach sample, we scanned it for unusual or large prey items, such as fishes, which we also put aside for analysis alongside the homogeneous content. Additional samples of stomach contents (*n* = 25, 28%) were opportunistically collected between February and June in 2013 and 2014, without dissecting or weighing the whole stomach.

**Table 1.** Sample sizes, disc width and weights of stomach contents. For each mobulid species, we present the disc width range, median and sample size in brackets for females and males. We also present the stomach contents weight range, mean and sample size in brackets for both sexes. Also shown are the sample sizes of all examined stomachs (taxonomy) and those available for quantitative species analyses (quantitative).

	disc width (cm)		stomach contents weight (g)		microscopy sample sizes	
species	females	males	females	males	taxonomy	quantitative
*Ma. birostris*	231–552, 515 (14)	38–446, 394 (10)	0–4408, 952 (10)	0–5050, 1080 (7)	19	6
*M. tarapacana*	195–242, 213 (5)	200–279, 255 (12)	12–368, 213 (5)	48–4307, 875 (12)	22	11
*M. japanica*	148–204, 181 (7)	154–232, 200 (12)	0–753, 252 (6)	20–608, 365 (11)	22	13
*M. thurstoni*	108–187, 163 (19)	126–182, 161 (22)	27–845, 335 (18)	0–860, 277 (21)	26	12

### 2.1. Microscopy

Stomach contents from *Ma. birostris* (*n* = 19), *M. tarapacana* (*n* = 22), *M. japanica* (*n* = 22) and *M. thurstoni* (*n* = 26) were visually examined under a dissecting microscope. This dataset included both sexes and immature and mature animals of all species. Some 2.5 ml samples contained few identifiable specimens and were thus analysed as a whole. Some of the samples contained many prey specimens and we, therefore, diluted samples in 50 ml of water and took 2.5 ml subsamples for quantitative analysis using a Stempel pipette. We counted prey specimens in a Bogorov tray until at least one full subsample was analysed, and if fewer than 30 specimens were counted, we examined further subsamples. Results from quantitative analyses are expressed as a percentage of total counts (%N). In addition to these quantitative counts, we scanned the whole sample to reveal unusual items not seen in the counted subsamples. To give a more complete view of their diet, we used these qualitative taxonomic data to produce a prey list for each mobulid species (table 2), expressed as per cent frequency (*F*_O_). As this prey list is based on qualitative data, we could not calculate per cent numerical occurrence (%N). All specimens were identified to the lowest possible taxon using keys [30]. As most of the specimens were in a digested state, we often counted heads, tails and eyes to estimate the abundance of a species. Their digested state also meant that we could not weigh prey items and most of the large and unusual prey items were only found in the examination of the whole sample, not the quantitative subsample, and thus we did not calculate a compound index as is usually done [31].

**Table 2.** Full prey list. All prey items found in stomachs of the four species of mobulid rays, with phylum, order and species, as well as the number of stomachs containing the prey and the percentage frequency of occurrence (%*F*_O_). Not included are parasitic trematodes, cestodes and copepods that were in the stomach, but not as prey.

			*n*	%*F*_O_
*Manta birostris* (*n* = 19)				
Arthropoda	Euphausiacea	*Euphausia diomedeae*	15	78.9
	Copepoda	*Labidocera detruncata*	2	10.5
	Copepoda	*Pontella cf tenuiremis*	2	10.5
	Copepoda	*Cosmocalanus darwinii*	1	5.3
	Copepoda	*Harpacticoida*	1	5.3
	Copepoda	*Onceaea venusta*	1	5.3
	Copepoda	*Pontella fera*	1	5.3
	Copepoda	*Pontella* sp.	1	5.3
	Copepoda	unidentified	1	5.3
	Ostracoda	unidentified	1	5.3
Chordata	Myctophidae	*Myctophum asperum*	1	5.3
	Myctophidae	*Myctophum spinosum*	1	5.3
Mollusca	Gastropoda	*Diacavolinia* sp.	1	5.3
	Gastropoda	unidentified gastropod shell	1	5.3
unidentified		egg	1	5.3
marine algae		marine algae	1	5.3
plants		seed	1	5.3
*Mobula tarapacana* (*n* = 22)				
Arthropoda	Euphausiacea	*Euphausia diomedeae*	18	81.8
	Copepoda	unidentified	1	5.6
Chaetognatha	Sagittoidea	unidentified	1	5.6
Chordata	Teleostei	unidentified	1	5.6
Mollusca	Teuthida	squid	1	5.6
unidentified		egg	2	11.1
*Mobula japanica* (*n* = 22)				
Arthropoda	Euphausiacea	*Euphausia diomedeae*	22	100.00
	Brachyura	unidentified larva	1	4.55
	Copepoda	unidentified	1	4.55
	Euphausiacea	unidentified krill	1	4.55
unidentified		egg	1	4.55
*Mobula thurstoni* (*n* = 26)				
Arthropoda	Euphausiacea	*Euphausia diomedeae*	26	100.0
	Copepoda	unidentified	1	3.8
unidentified	unidentified	polychaete larvae	1	3.8
plants		seed	1	3.8

For six *Ma. birostris*, 11 *M. tarapacana*, 13 *M. japanica* and 12 *M. thurstoni*, we had both the stomach contents weight and the proportion of the whole stomach content that was analysed. This subset allowed us to quantify prey in a whole stomach. For example, a *M. japanica* had a stomach contents weight of 554 g of which we collected 3.85 g for microscopy analysis. We diluted that subsample in 50 ml of water and counted 2.5 ml of that in the Bogorov tray, equalling 0.035% of the total stomach contents.

### 2.2. Size spectrum and energy content

To determine the absolute and relative biomass as well as the size spectrum of mobulid prey, we used a 2400 dpi resolution ZooScan [32] on 14 selected samples (*Ma. birostris n* = 5, *M. tarapacana n* = 2, *M. japanica n* = 3, *M. thurstoni n* = 3) that contained the least-digested specimens. Formalin-preserved samples were processed according to scanning methods outlined in Schultes & Lopes [33]. To reduce the incidence of touching particles, prey items were physically separated in the scanning tray, as well as digitally separated after the scan. Plankton identifier software was used to ensure accurate classification of whole organisms versus parts of organisms (e.g. euphausiid legs) and scanning artefacts (bubbles, shadows) [32]. Non-whole organisms and detached organism body parts, as well as scanning artefacts were subsequently removed from the analysis. The particle pixel area measurement was converted to square millimetres and this was used to estimate spherical biovolume (SBv). The normalized biomass size spectra were then calculated by summing the SBv of each particle into 50 normalized size bins. Zooscan-calculated length (millimetres) of stomach content particles was used to create a prey size spectrum for each mobulid species. The log-transformed SBv was used to compare stomach content particles from this study with particle size from zooplankton tows taken during other planktivorous elasmobranch feeding events from Mafia Island in Tanzania and Lady Elliot Island in Australia [18,19]. The maximum particle length and SBv is reported alongside the mean as it represents the least-digested and intact prey particles.

Gross energy of *Euphausia diomedeae* and *Myctophum asperum* was determined by adiabatic bomb calorimetry [34] using a Parr 6200 Bomb Calorimeter. Gross energy is expressed either as kcal or kJ g^−1^ dry weight. To compare our values with the literature, we derived an approximate correction factor (1.144) for formalin-stored samples by measuring gross energy of krill meal (Norwegian krill meal, Ridley AquaFeed) preserved in formalin (*n* = 1; 33.22 kJ g^−1^) and raw (*n* = 1; 37.99 kJ g^−1^). Using the quantitative stomach contents samples, we scaled the calorific values of *E. diomedae* to kJ/100 g of stomach content to compare the energetic input of this prey among the four mobulid species. We also scaled the calorific value of *E. diomedeae* to total kJ per whole stomach contents to assess energy intake from this prey species. Data are available on Dryad [35].

## 3. Results

### 3.1. Fished mobulids

Four mobulid species were regularly landed during our sample collection: *Ma. birostris*, *M. tarapacana*, *M. japanica* and *M. thurstoni*. For all species, individuals examined in this study were from both sexes and from a mix of juveniles and adults. Multiple species spanning a range of sizes and sexes were often caught in the same net. Fishing locations were mostly over oceanic areas > 1000 m deep, although some catches were also made in areas between 200 and 1000 m depth (figure 1).

### 3.2. Stomach and stomach contents weights

**Figure 2.**
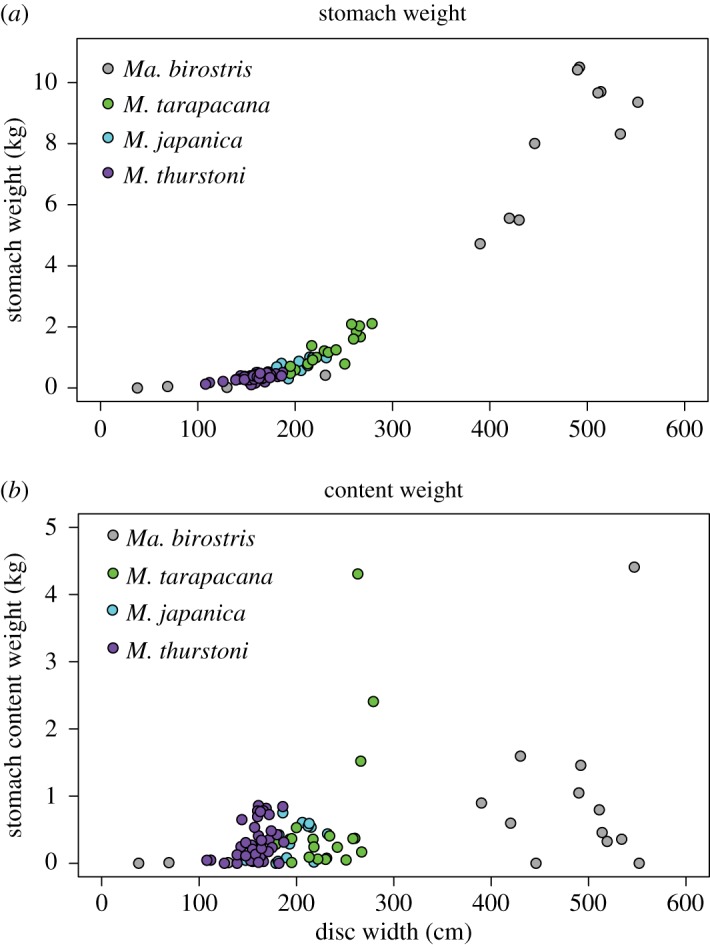
Stomach and stomach content weights. Plots of animal disc width and (*a*) empty stomach weights and (*b*) stomach content weights for the four mobulid species.

*Manta birostris* was the largest species at up to 552 cm disc width (table 1), with heaviest stomachs and stomach contents of up to approximately 5 kg, followed by *M. tarapacana*, *M. japanica* and *M. thurstoni* (figure 2*a*). Larger animals had larger stomachs and, therefore, a higher capacity for food intake. However, several animals had empty or near-empty stomachs, resulting in a more variable relationship between stomach contents weight and animal size (figure 2*b*).

### 3.3. The major prey: *Euphausia diomedeae*

Stomach contents of all mobulid species were dominated by the krill *E. diomedeae* with 91% of stomachs containing the species (*n* = 89) and 93% of all identifiable prey items were *E. diomedeae*. While whole specimens were lacking from some stomach contents samples that were too digested, these samples contained at least some diagnostic fragments (antennae, carapace, rostrum and eyes) that allowed species identification. These features included a bifurcate lappet on the first segment of the antenna, spines on the second segment of the antenna, an acute rostrum extending to the anterior limit of the eye, and two denticles on the carapace. Intact specimens of *E. diomedeae* were adults, although some of the separated digested parts, such as eyes or rostra, could also have come from larvae. Of the 19 *Ma. birostris* stomachs examined, 14 (*F*_O_ = 79%) contained *E. diomedeae*, which was the lowest frequency of occurrence of *E. diomedeae* in the four mobulid species (table 2). Three *Ma. birostris* stomachs were empty and one stomach contained no krill but a few copepods. The majority of *M. tarapacana* stomachs contained *E. diomedeae* (*F*_O_ = 82%), with four of 22 containing no identifiable prey items. *Mobula japanica* and *M. thurstoni* had krill in 100% of stomachs (table 2).

Quantitative analyses of the counted subsamples (*n* = 42) showed that 93% of all counted prey items overall were *E. diomedeae*. The %N of *E. diomedeae* was lowest in *Ma. birostris* (mean = 68%, range = 0–100%) that had one stomach containing copepods without krill, followed by *M. tarapacana* (mean = 96%, range = 64–100%), *M. japanica* (mean = 99%, range = 93–100%) and *M. thurstoni* (mean = 100%).

### 3.4. Energy content

**Figure 3.**
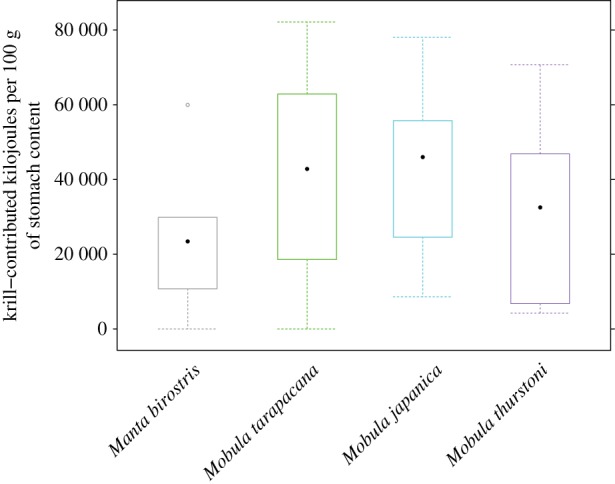
Calorific contribution of krill per 100 g of stomach content. Calorific content of all *E. diomedeae* from counts in subsamples extrapolated to 100 g of stomach content for the four mobulid species.

The energy content of one sample of *E. diomedeae* was 19.4 kJ g^−1^ dry weight, or 2.42 kJ per organism. Scaling the energy content up to the total number of *E. diomedeae* per stomach contents weight showed that up to 82 000 kJ/100 g of stomach contents was contributed by krill (figure 3). The mean number of individual krill per volume, and thus the calorific value per 100 g, were similar among the four species, although the standard deviation was large. Krill made up a variable caloric contribution to 100 g of stomach content, ranging from a minimum mean of 24 572 kJ (±20 451 kJ s.d.) in *Ma. birostris* to a maximum mean of 40 760 kJ (±20 048 kJ s.d.) in *M. japanica*.

The myctophid fish *My. asperum* had a calorific value of 16.0 kJ g^−1^ dry weight and 39.8 kJ per organism. The calorific value was similar on a per gram basis to *E. diomedeae*, but due to its larger size, the per individual calorific value was approximately 16 times higher for the myctophid fish. Its contribution to total energy intake was minimal, however, because we only found two fishes in our samples.

### 3.5. Size spectrum and biovolume of euphausiids

**Figure 4.**
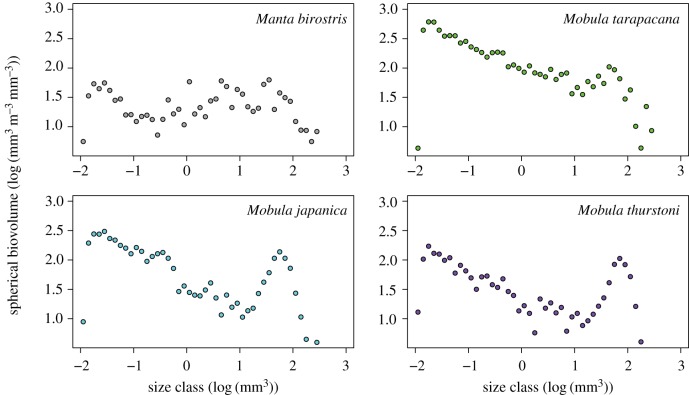
Normalized biovolume size spectra of *Euphausia diomedeae* from mobulid stomach contents determined using ZooScan.

**Figure 5.**
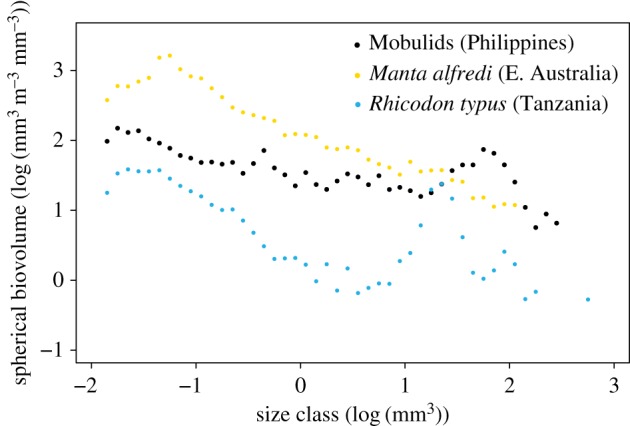
Mean normalized biovolume size spectra of *Euphausia diomedeae* in mobulid stomach contents collected in the Philippines. Also included are feeding samples from *in situ* zooplankton tows for *Manta alfredi* [18] and *Rhinocodon typus* [19].

There was no significant difference in the mean (ANOVA, *F*_3,8_ = 0.128*, p *= 0.94) or maximum (ANOVA, *F*_3,8_ = 3.095*, p *= 0.09) spherical biovolumes (SBv_P_) of zooplankton particles in stomach contents among species. There was also no significant difference in the mean (ANOVA, *F*_3,8_ = 0.116*, p *= 0.95) or maximum (ANOVA, *F*_3,8_ = 0.218*, p *= 0.88) body length of zooplankton particles in stomach contents among different mobulid species (figure 4, table 3). Among all four mobulid species, maximum particle lengths ranged from 13.7 to 14.1 mm (table 3). There was a distinct peak in larger body volume sizes for particles collected from mobulid stomachs overall (figure 5). This peak in larger prey particles in the biovoulme distribution was especially apparent in stomach contents of *M. japanica* and *M. thurstoni* (figure 4). Comparing our results with the sizes of prey items determined from zooplankton collected during surface feeding events elsewhere shows that whale shark feeding samples similarly occurred when individual prey size was larger than in background samples, while *Ma. alfredi* samples had no such peak during feeding events (figure 5).

**Table 3.** ZooScan measurements. The maximum (±s.e.) zooplankton particle biovolume (SBv_P_) and zooplankton particle length found in the stomach contents of each mobulid species, with the literature values of other planktivorous elasmobranchs as comparison. Sample types include stomach contents (SC) and zooplankton tows during feeding events (Tow). SB_VT_ of stomach contents (per litre of stomach contents) and zooplankton tows (per litre of filtered water) are not directly comparable.

species	sample type	SBv_P_ (mm^3^)	length (mm)	reference
*Ma. birostris*	SC	17.8 ± 2.9	3.5 ± 0.3	this study
*M. tarapacana*	SC	16.5 ± 3	3.4 ± 0.3	this study
*M. japanica*	SC	19.7 ± 3.7	3.4 ± 0.4	this study
*M. thurstoni*	SC	15.7 ± 3.2	3.4 ± 0.4	this study
*Ma. alfredi*	Tow	0.4 ± 0.2	1.1 ± 0.1	[18]
*Rhinocodon typus*	Tow	7.3 + 0.9	3.7	[19]

### 3.6. Other prey items

Prey other than *E. diomedeae* had a low incidence, with the frequency of occurrence ranging from 3.8 to 11%. *Manta birostris* had the highest prey diversity, including two species of myctophid fishes (*My. asperum* and *My. spinosum*), several species of copepods, an ostracod, *Diacavolinia* gastropod, unidentified eggs and some algae and a plant seed (table 2). One specimen contained five individual *My. asperum* among the ubiquitous soup of krill, while the other myctophid was a single specimen. *Mobula tarapacana* had a squid beak and some squid flesh in one stomach and a piece of unidentifiable fish in another. One *M. japanica* stomach contained crab larvae, and copepods were found in *M. japanica* and in *M. thurstoni* (table 2).

## 4. Discussion

*Euphausia diomedeae* was the dominant prey in terms of abundance and incidence in the stomach contents of all mobulids in this study. The strong dietary overlap in all mobulid species here suggests that in the Bohol Sea during November–May, *E. diomedeae* is not a limited resource. The prevalence of *E. diomedeae* in this region probably provides a mechanism that facilitates the coexistence and thereby limits interspecific competition among these sympatric mobulid species. *Mobula japanica* and *M. thurstoni* almost exclusively fed on this krill species. By contrast, *Ma. birostris* had a broader dietary intake, also including a few fishes and copepods, supporting biochemical results that found there are large variations in the diet between *Ma. birostris* individuals within the same subpopulation [17]. Stomach contents provide a short-term snapshot of a species' diet [36], and while we sampled several species over several months and found *E. diomedeae* to be the major prey item throughout, we could not ascertain what these rays feed on when they are not getting caught in surface waters, when they are not in the Bohol Sea, or outside the November–May period. Longer-term dietary information from biochemical analyses could provide a more complete temporal view of dietary intake for these species.

### 4.1. The major prey item: *Euphausia diomedeae*

*Euphausia diomedeae* is distributed through the Indo-Pacific in warm oceanic waters and is a common species in the Philippines [30,37]. Euphausiids form swarms to feed or spawn [38], making them a good target for large filter feeders. Mobulids were caught during November–May, which coincides with an intensification of upwelling in the Bohol Sea [22,23]. This increase in upwelling in the Bohol Sea probably plays a major role in the seasonal abundance of *E. diomedeae*, which then attracts large numbers of feeding mobulids.

All four species of mobulids were caught together at night, sometimes in the same net, showing that they were in the same area together, which strongly suggests they were feeding as opposed to mating or socializing. Although it is possible that mobulids in the Bohol Sea feed on *E. diomedeae* during the day and get caught at night when transiting between feeding areas, it is more likely that they target them in shallow waters < 35 m at night, where they were caught by the fishers. First, stomach contents from all species overall contained intact specimens despite most rays being dead for several hours prior to dissection, suggesting that the krill were recently ingested. Second, similar to other euphausiids [39], *E. diomedeae* migrate vertically, staying deep during the day (140–280 m layer) and shallow, including at the surface, at night [37], which is when and where the mobulids were caught. *Euphausia diomedeae* reproduce most heavily in May in the nearby South China Sea, although larvae and adults there were abundant at all times [30]. They shed their eggs into the water column after fertilization [30] and adults and juveniles may thus not stay together, which could help explain why we found adults but no larvae in the stomachs of mobulid species. However, it is also possible that larvae get digested faster than adults due to their smaller size. Mobulids preferentially filtering the smaller larvae out is less likely, as we also found relatively small eggs and copepods in the stomachs, but no larvae.

A central assumption of stomach content studies is that samples are representative of a species' prey preference. However, this may not always be the case due to differential prey residency times in the gut [40]. Other prey items may have been digested faster than *E. diomedeae*, resulting in an overstated importance of this krill species to the diet of mobulid species in the Bohol Sea. Gelatinous zooplankton, in particular, could have been missed in our stomach contents analysis.

### 4.2. Mobulid prey composition

Euphausiids were also the dominant prey in *M. thurstoni* and *M. japanica* from the Gulf of California [4] and in one *M. thurstoni* stomach from the Western Atlantic [41]. Euphausiids are also a common prey item of a multitude of other planktivorous species including baleen whales [42,43], whale sharks *Rhincodon typus* [44], megamouth sharks *Megachasma pelagio* [45] and mobulids [4]. Although *E. diomedeae* was only found as prey in mobulids in the present study and for Bryde's whales in Madagascar [46], the exact species of euphausiid is unlikely to be of importance to their predators. Their large (10–15 mm) size relative to other zooplankton, and swarming behaviour makes them a good target for large filter feeders, such as mobulid rays. Many other large filter feeders also target large-bodied zooplankton prey in high densities. In *Ma. alfredi*, an historical stomach contents sample from Eastern Australia contained calanoid copepods predominantly, which were at the large end of the size spectrum for regional zooplankton [10]. Similarly, whale sharks in Tanzania targeted dense patches of large sergestids in a coastal area otherwise dominated by smaller copepods [19] and basking sharks, *Cetorhinus maximus*, in England fed in areas dominated by larger copepod species compared to where basking sharks were absent [47]. This trend to large-bodied prey is not uniform, however, as *Ma. alfredi* targeted dense patches of small copepods in eastern Australia [18]. This recent example suggests that individual prey size itself may not be important as long as the prey biomass is high. Energetically, it makes sense for filter feeders to feed when biomass of catchable zooplankton is high, irrespective of the taxonomic composition or size of individual particles.

The mean size and particle biovolume SBv_P_ of prey here was larger compared to zooplankton collected from surface feeding events in other planktivorous elasmobranch feeding studies [18,19]. These previous studies were conducted in coastal areas, and our data from mobulids caught in the open ocean indicate that prey here was larger and potentially in more highly concentrated patches. Mesopelagic prey that vertically migrate from below 200 m during the day to surface waters, such as *E. diomedeae* here, could be an important food source for filter-feeding elasmobranchs, in general. Vertically migrating mesopelagic prey are abundant [48,49] and filter feeders in the low latitudes, where surface waters are generally oligotrophic [50], need prey sources other than the low densities of daytime surface zooplankton to obtain sufficient food. They do not need to dive deep to access this resource, since many of their mesopelagic prey vertically migrate to shallow waters at night. *Manta birostris*, *M. tarapacana*, *M. mobular* and whale sharks have been suggested to feed on mesopelagic prey based on vertical movement data and fatty acid analysis of tissue samples [14,26,28,51]. Here, we show directly that mobulids feed on mesopelagic prey, adding to the evidence that this food source is important for large filter feeders in the tropics.

### 4.3. Trophic separation

We found a surprising lack of trophic separation in the short-term diet of the four mobulid species in the Bohol Sea. Trophic separation is a common mechanism by which competing species coexist, resulting in functionally similar species evolving dissimilar diets and thereby alleviating interspecific competition [52]. For example, sympatric benthic batoid ray species have different jaw morphologies that are linked to different efficiencies in capturing different prey types [53]. Species in the family Mobulidae have slightly different gill raker feeding structures [54], and differ considerably in size, suggesting that, although all are filter feeders, there may be differences in the prey species and sizes. However, all species here fed heavily on *E. diomedeae*.

While *E. diomedeae* dominated the stomach contents of all four species, the larger two had a few additional prey items, including myctophid fishes in *Ma. birostris* and fish and squid in *M. tarapacana*. These larger prey items may be evidence of a small degree of trophic separation in mobulids, or simply representative of opportunistic energy subsidation in the absence of high-density krill patches. It is possible that the larger mobulids can dive deeper and thus access additional prey sources compared to the smaller *M. japanica* and *M. thurstoni*. Larger rays have a larger thermal inertia and may thus be able to tolerate cooler, deeper waters for longer than smaller rays. *Mobula tarapacana* in the Azores [26] and *M. mobular* with a disc width of 2.5–3 m in the Mediterranean [51] dived to greater than 1500 m and 700 m, respectively. *Manta birostris* in Mexico shifted to spending more time in the deep scattering layer during the second half of the calendar year [28]. The smaller *M. japanica*, although diving to a maximum of 1112 m, only occasionally ventured deeper than 200 m and spent most time above the deep scattering layer [27,55]. It is, therefore, likely that the larger mobulids have a wider vertical range and thus different feeding options than smaller species. However, the myctophid species in *Ma. birostris* stomachs are diurnal vertical migrators [56,57] and many squid species also occupy surface waters at night [58], meaning that dive ability may not explain these specific additional prey items in the larger two mobulid species. It is also possible that the larger *Ma. birostris* and *M. tarapacana* can swim faster to catch fast-swimming prey, or that their larger mouth opening negates the flight behaviour of prey in close proximity.

In the absence of trophic separation, sympatric elasmobranch species may coexist when they separate in time and space [59,60]. Mobulids here were caught over deep oceanic waters, often together in the same net with catches comprising males, females, juveniles and adults of all four species, which range in size from approximately 1 m *M. thurstoni* to a 5.5 m wide *Ma. birostris*. This contrasts with temporal size segregation seen in *M. thurstoni* in Mexico [4] and the generally observed sexual segregation in elasmobranchs [61]. The lack of separation in diet and space use here indicates that prolific pulses of *E. diomedeae* in the Bohol Sea during November–May alleviate competition between mobulids, which results in large trophic and habitat overlaps among these species.

### 4.4 Energetics

The energy contents of prey from mobulid stomachs were largely similar to the literature values, suggesting that mobulid stomach content samples can potentially be used for energy budget calculations. The major caveat is that the time frame over which the predators acquired their prey is not known. *Euphausia diomedeae* at 19.1 kJ g^−1^ had slightly lower calorific values than Antarctic *E. superba* (approx. 22 kJ g^−1^; [62,63]). The myctophid fish *My. asperum* at 16.0 kJ g^−1^ had lower energy content than myctophids in the sub-antarctic (approx. 26 kJ g^−1^; [64,65]). It also had a lower calorific value per gram than *E. diomedeae* in our study, but a single myctophid contains much more energy than a euphausiid (39.8 versus 2.4 kJ), due to their larger mass.

Scaling the total number of euphausiids per stomach content showed that *E. diomedeae* contributed up to 631 167 kcal in a *Ma. birostris* stomach content, although medians ranged between 25 343 kcal in *M. thurstoni* to 50 365 kcal in *M. japanica*. This is more than expected, considering that captive *Ma. alfredi* are fed 3500 and 6100 kcal per day in aquaria (disc width = 350 and 450 cm; A. Dove 2016, personal communication) and whale sharks in Mexico had an estimated intake of 3570 and 6720 kcal per day (total length = 443 and 622 cm; [66]). These numbers indicate that a mobulid stomach in our study may contain the krill remains of many feeding events over several days. Alternatively, mobulid species may feed sporadically on large pulses of prey when available and then go through a starvation period until they find their next meal. This hypothesis could account for the large differences between the calculated calorific value of stomach contents versus what *Ma. alfredi* are fed daily in aquaria.

Many individuals also had empty or near-empty stomachs, especially in the larger species *Ma. birostris* and *M. tarapacana*. It is possible that empty stomachs were a consequence of stomach eversion due to capture stress. This is unlikely, however, because buccal cavities were intact in sampled specimens, indicating that stomach eversion had not recently occurred, although rays are capable of swallowing their stomachs after eversion [67]. In the Gulf of California, the majority of stomachs from *M. thurstoni*, *M. japanica* and *M. tarapacana* were empty [4]. The prevalence of empty stomachs across all sampled mobulid species in different regions adds weight to the hypothesis that these species go through cycles of starvation and large feeding events. Those individuals with empty stomachs are likely to have only recently moved into the area to capitalize on the abundant krill resource, but had not done so before being captured. Such a boom and bust feeding behaviour is common in predatory elasmobranch species [68,69], although the prevalence of this behaviour in planktivorous elasmobranchs has not been investigated. Mobulids and other tropical planktivorous elasmobranchs live in comparatively oligotrophic environments [50], where zooplankton is patchily distributed in space and time [70], and large feeding events followed by a period of starvation is, therefore, a likely adaptation needed to survive here.

## Data accessibility

Data are available on Dryad: (http://dx.doi.org/10.5061/dryad.g75j9) [35].
